# Mixed Waste Streams for Bioproduction: Exploring Bacterial Wax Ester Production in Nitrogen‐Rich Acidogenic Fermentate

**DOI:** 10.1111/1751-7915.70314

**Published:** 2026-02-16

**Authors:** Laura K. Martin, Wei E. Huang, Ian P. Thompson

**Affiliations:** ^1^ Department of Engineering Science University of Oxford Oxford UK

**Keywords:** acidogenic fermentation, anaerobic digestion, bioproduction, glyoxylate shunt, microbial lipids, volatile fatty acids, wax esters

## Abstract

Microbial lipids offer a promising alternative to petrochemicals, but high associated costs and low conversion efficiencies pose barriers to their commercialisation. In particular, sugar‐based feedstocks are too expensive for the production of commodity chemicals, and recently attention has turned to volatile fatty acids (VFAs) as a cheaper, more widely available carbon source. Acidogenic fermentation can be used to produce high concentrations of VFAs from municipal and agricultural waste. By harnessing metabolically engineered *Acinetobacter baylyi* ADP1, the suitability of VFAs as sole carbon sources for wax ester (WE) production was investigated. These studies resulted in the highest WE accumulation in ADP1 achieved to date, at 37% of cell dry weight, and the first reported production of bacterial WEs from a raw, mixed waste stream, utilising fermentate as the sole carbon source. WE titres of over 160 mg/L from VFAs were achieved, highlighting the unique benefits of mixed feedstocks typically considered problematic for bioproduction. Finally, the potential advantages of employing fermentates rich in longer chain VFAs are explored. In synthetic media, WE titres up to 190 mg/L were achieved, but translation to fermentate was challenging, emphasising the need for continued research in this area.

## Graphical abstract

The bacterium *Acinetobacter baylyi* ADP1, a known source of biogenic wax esters, and its glyoxylate shunt knockout mutant, were demonstrated to grow on short‐chain fatty acids and directly on acidogenic fermentate from arrested anaerobic digestion of biomass. Compositional control of the fermentate results in different growth and wax accumulation behaviour.

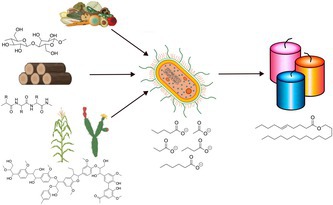

## 1 Introduction

Microbial storage lipids have been identified as an attractive, renewable alternative to a variety of petrochemical compounds including polymers (Rodriguez‐Perez et al. 2018), fuels (Koreti et al. 2022; Shields‐Menard et al. 2018) and waxes (Martin et al. 2021). However, the production of storage lipids is inherently in competition with growth at the metabolic level (Luo et al. 2020). Nutrient limitation can overcome a cell's natural tendency to direct resources to growth, instead shifting carbon flux towards storage lipids (Castro et al. 2018; Manilla‐Pérez et al. 2011; Martin et al. 2023), but this comes at a cost; growth is restricted and, ultimately, production is limited.

To circumvent this problem, recent advances in metabolic engineering have attempted to balance growth and bioproduction by controlled co‐feeding of two distinct substrates to concurrently activate both pathways. This can be achieved by exploiting multiple naturally‐occurring metabolic routes (Park et al. 2019), or by engineering a partition of central carbon metabolism such that the pathways for growth and bioproduction are decoupled (Luo et al. 2020; Santala et al. 2018). Such approaches are particularly attractive if a cheap carbon source, used as the major production‐supporting substrate, is ‘doped’ with small amounts of rich growth‐supporting substrate. This reduces reliance on expensive, sugar‐based feedstocks and results in moderate growth coupled with efficient lipid production. This approach recently achieved wax ester (WE) production with a total C/C yield of 18%, the highest ever reported, in engineered *Acinetobacter baylyi* ADP1 co‐fed acetate and small amounts of glucose (Santala et al. 2021). Acetate is a popular, low‐cost, production‐supporting substrate due to its potential to be sourced from waste streams (David et al. 2020; Lehtinen et al. 2017; May et al. 2016) and the direct conversion of acetate to acetyl‐CoA, directly upstream of the fatty acid biosynthesis pathway, makes it a logical substrate for lipid production (Gong et al. 2015).

One abundant, sustainable source of acetate is acidogenic fermentation (AF), an arrested anaerobic degradation process widely used to treat organic waste including post‐consumer food (David et al. 2020; Dahiya et al. 2015; Srisowmeya et al. 2020), agricultural (Iglesias‐Iglesias et al. 2021) and sewage wastes (Ramos‐Suarez et al. 2021; Tayou et al. 2022). The fermentate comprises a mix of the volatile fatty acids (VFAs) acetate, propionate, butyrate, valerate and hexanoate and the exact VFA composition can be altered significantly by varying fermentation conditions including substrate carbon:nitrogen (C:N) ratio, pH, temperature, organic loading, initial inoculum, and retention time (Gong et al. 2021; Moretto et al. 2019; Tenci et al. 2023). For example, increasing the AF temperature can increase the proportion of butyric acid generated (Gong et al. 2021), whereas increasing the pH favours shorter chain VFAs acetic acid (Gong et al. 2021) or propionic acid (Atasoy and Cetecioglu 2022) depending on substrate and conditions. The proportion of longer chain fatty acids has been widely observed to increase, and conversely the proportion of acetate decrease, when solid or hydraulic retention time increases (Tenci et al. 2023; Yuan et al. 2009).

Fermentate has been utilised by a number of researchers for microbial lipid production, predominantly polyhydroxyalkanoates (PHAs) (Papa et al. 2020; Duque et al. 2014; Kumar et al. 2016; Aremu et al. 2021; Szacherska et al. 2021) and fatty acids for biodiesel (Annamalai et al. 2020; Patel et al. 2021). PHA production from waste derived VFAs has been documented in mixed microbial cultures (Papa et al. 2020; Duque et al. 2014), as well as by individual species of *Bacillus* (Kumar et al. 2016), *Pseudomonas* (Aremu et al. 2021) and *Cupriavidus* (Szacherska et al. 2021). PHA contents of up to 67% of CDW have been reported from the fermentation of low nitrogen waste streams such as wastepaper and molasses, whereas feedstocks richer in nitrogen (such as municipal waste) typically result in lower PHA accumulation, in the range of 30%–50% (Aremu et al. 2021; Szacherska et al. 2021). By contrast, for biodiesel production, research has predominantly focussed on pure culture fermentations as in bacteria long chain lipids are a less common energy storage molecule than PHAs (Wältermann and Steinbüchel 2005). Accumulation is also typically lower, with maximum reported lipid contents around 40% (Annamalai et al. 2020), due to the more reduced nature of the products and the longer synthesis route required: PHAs are produced from the polymerisation of 3‐hydroxy‐butyric and ‐valeric acids, so synthesis from VFAs is relatively simple, whereas long chain fatty acids require de novo synthesis from acetyl‐CoA and NADPH. For these reasons, long chain lipid production from VFAs is relatively more challenging and less investigated, making WEs a novel and interesting target for bioproduction from this substrate. Furthermore, despite its broad tunability, only a handful of studies have explored how VFA composition can influence the yield or composition of microbial lipids (Gong et al. 2021; Kumar et al. 2016; Al Battashi et al. 2021; Albuquerque et al. 2011).

**FIGURE 1:**
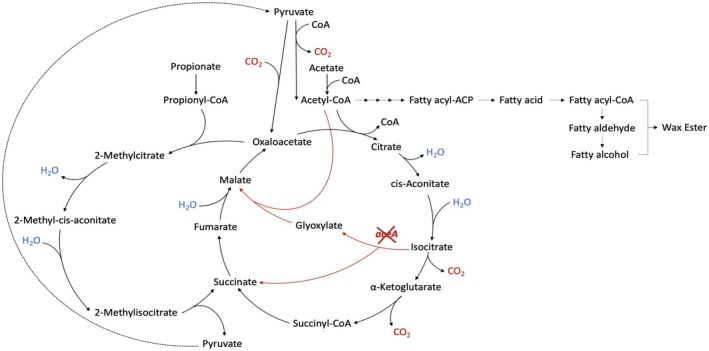
Map of the interconnected acetate and propionate metabolism cycles in *A. baylyi* ADP1. The *aceA* knock‐out in the glyoxylate shunt is shown: *aceA* is essential for bacteria to utilise two‐carbon feedstocks, such as acetate, for growth and the production of carbohydrates. Without *aceA*, cells cannot convert acetate into biomass, although it can be used for energy generation and to produce bioproducts such as wax esters.

The scope of this study was to demonstrate that the multiple components of an untreated, mixed waste stream, which are commonly considered a hindrance to biological chemical processes, could intrinsically provide co‐feeding of multiple substrates and hence yield advantages for individually controlling growth and lipid production. Using *A. baylyi* ADP1 wild type (WT) and a previously engineered glyoxylate shunt knock‐out ADP1 M+ (Figure 1), VFAs were individually investigated for their suitability as sole carbon sources, showcasing the utilisation of several substrates not previously demonstrated in ADP1. The effect of genetic engineering in M+ on singular and co‐feeding substrate tolerance was explored and the relative proportions of each substrate varied to supply carbon to growth and production pathways at different rates. The first example of WE production from VFA rich fermentate of comparable composition was then attempted, with production significantly increased in the M+ strain over the WT bacteria.

Due to the inherent acid tolerance limit of ADP1 observed during this work, the utilisation of longer chain VFAs was also explored to increase available carbon density and reducing power without increasing total acid concentration. Following successful results with pure butyrate and valerate, ADP1 WT and M+ were grown on fermentate rich in these acids. The ability to generate such diverse fermentate compositions exemplifies the benefits of fermentate tuneability for microbial growth and bioproduction. However, translation of the results under this condition was not trivial, emphasising the difficulty of replicating results from synthetic laboratory media on complex, mixed substrates. Overall, our findings here are applicable to the valorisation of a wide range of organic waste streams.

## 2 Materials and Methods

### 2.1 Bacterial Strains, Chemicals and Culture Media

Stocks of *A. baylyi* ADP1 (DSM 24193) wild type and *A. baylyi* Δ*acr1*::*Kan* ^*r*^ */tdk* Δ*aceA*::P_T5_‐*acr1‐spec* ^*r*^ (ADP1 M+) (Luo et al. 2020) (kindly donated by V Santala, Tampere University, Finland) were stored at −80°C in 20% v/v glycerol/water. All chemicals sourced from Sigma‐Aldrich (Merck).

### 2.2 Culture Conditions

Glycerol stocks were streaked on LB agar plates and incubated at 30°C for 24 h. Single colonies were picked and inoculated into LB broth, grown to mid‐exponential phase and transferred to precultures in flasks either LB broth or minimal media. Minimal media contained 2.5 g/L KH_2_PO_4_, 2.5 g/L Na_2_HPO_4_, 1 g/L NH_4_Cl, 0.1 g/L MgSO_4_·7H_2_O, 0.01 g/L CaCl_2_·2H_2_O and 1 mL/L of 1000× Bauchop and Elsden trace elements solution (Bauchop and Elsden 1960). Medium was adjusted to pH 6.8 and autoclaved. 300 g/L sodium acetate, 300 g/L sodium propionate, 100 g/L sodium butyrate, 40 g/L valeric acid and 10 g/L hexanoic acid stocks were adjusted to pH 6.8, autoclaved, and added prior to inoculation. ADP1 M+ cultures and plates contained kanamycin and spectinomycin at 50 μg mL^−1^. Immediately prior to each experiment, precultures were spun down at 6000 g for 6 min, resuspended in phosphate buffer and experiments were inoculated. All liquid cultures and precultures were cultivated at 30°C and 150 rpm.

### 2.3 Plate Reader Experiments

VFA tolerance experiments were performed in clear‐bottomed, black 96‐well plates on a Tecan Spark (Tecan Group Ltd., Männedorf, Switzerland). Each well contained 200 μL of culture. Plates were incubated at 30°C at 180 rpm orbital shaking with 3 mm amplitude and 15‐min sampling interval time. A humidity cassette was used to prevent evaporation.

### 2.4 Wax Extraction and Quantification

Wax production experiments were conducted in 50 mL culture in 250 mL flasks or 200 mL culture in 1 L flasks. Growth was measured by OD_600_ with UV‐1800 UV/Vis spectrophotometer (Shimadzu, Kyoto, Japan). For NMR quantification, lipids were extracted from 10 to 25 mL of culture following an adapted Bligh and Dyer method as detailed previously (Martin et al. 2023; Santala et al. 2011). NMR analysis was performed as detailed previously (Martin et al. 2023). WE concentration was calculated from the triplet peak at *δ* 4.05 ppm, corresponding to the 2 α‐alkoxy methylene protons. WE mass was estimated assuming an average *M*_w_ of 506 g/mol (Lehtinen et al. 2018).

### 2.5 Acidogenic Fermentation

#### 2.5.1 *Opuntia* Digestion

*Opuntia ficus indica* cladodes were obtained from Pepe Aromas (Azaruja, Portugal). Cladodes were diced, dried at 60°C until a constant mass (5 days), ground in a blender (Waring, USA) and passed through a 1 mm screen. Fermentations were performed in 300 mL cultures in 500 mL glass bottles. A total of 200 mL/L of sludge and 39 g/L of volatile solids (VS) equivalent of opuntia were used, giving a substrate:inoculum ratio of 8:1 on a VS basis.

#### 2.5.2 Butyrate and Valerate Enriched Digestion

Food‐grade dried pig blood was obtained from Tongmaster Seasonings (Coatbridge, UK). Fermentations were performed in 1 L cultures in 2 L bottles, with 330 mL/L of sludge and 60 g/L VS equivalent of dried blood.

#### 2.5.3 Inoculum and Media

Anaerobic digestion sludge from a local mesophilic food waste processing facility (Severn Trent Green Power, Cassington, UK) was used as an inoculum. Sludge was stored under anaerobic conditions at 30°C until use and deployed within 2 days of collection. Synthetic basic medium was prepared according to the literature recommendations, with the omission of sodium sulphide, to provide the necessary micronutrients, trace metals and vitamins (Angelidaki and Sanders 2004). The liquid reaction mixture (containing the basic medium, deionised water and sludge) was prepared in bulk, adjusted to pH 8.0 and added to bottles containing dried biomass. Bottles were incubated at 37°C and agitated at 125 rpm. Fermentations were incubated for 20 days, then extracted fermentate was stored at −80°C to prevent degradation.

### 2.6 Acidogenic Fermentate Growth and Wax Production

Fermentate was defrosted overnight at 4°C, centrifuged for 15 min at 5800 *g* and for 10 min at 10,000 *g*, pH adjusted to 6.8 then autoclaved. Post‐autoclaving, fermentate was re‐clarified by centrifugation for 10 min at 10,000 *g*, then stored at 4°C for up to 3 days before use. Fermentate was diluted to concentrations of 4–12 g/L of total VFA with deionised water, as required, and the final culture medium was autoclaved in flask prior to inoculation.

### 2.7 Volatile Fatty Acid Quantification by GC‐FID

VFAs were quantified using gas chromatography–flame ionisation detection (GC–FID) as detailed previously (Tenci et al. 2023). Calibration was performed with standards of acetic, propionic, isobutyric, butyric, isovaleric, valeric and hexanoic acids from 0.31 to 20.00 g/L. C‐conversion efficiency was calculated from the VFA consumption at the given time point, accounting for both the concentration and number of carbons in each acid. Theoretical maximum yield was calculated by balancing consumed VFA with the direct carbon incorporation and the utilisation of VFAs to generate necessary reducing equivalents and ATP. An average chain length of 34 carbons, requiring 32 molecules of NADPH, was assumed, with 2, 3, 4, 5 and 6 carbon fatty acids able to generate 2, 3, 6, 7 and 10 molecules of NADH, respectively, in their complete oxidation.

### 2.8 Statistical Analysis

Results were analysed for statistical significance in GraphPad Prism (GraphPad, San Diego, CA, United States). The maximum titre, content and C‐conversion was identified for each condition over the whole time‐course, and each set of maxima were analysed by one‐way ANOVA, with multiple pairwise comparisons. The Šidák correction was applied to control the false positive rate, and the Brown‐Forsythe test was performed to confirm that group variances were not significantly different (and hence that the data were suitable for ANOVA). Asterisks indicate significance at different levels as follows: * = 5%, ** = 1%, *** = 0.1% and **** = 0.01%.

## 3 Results

### 3.1 Volatile Fatty Acids as Sole Carbon Sources

**FIGURE 2:**
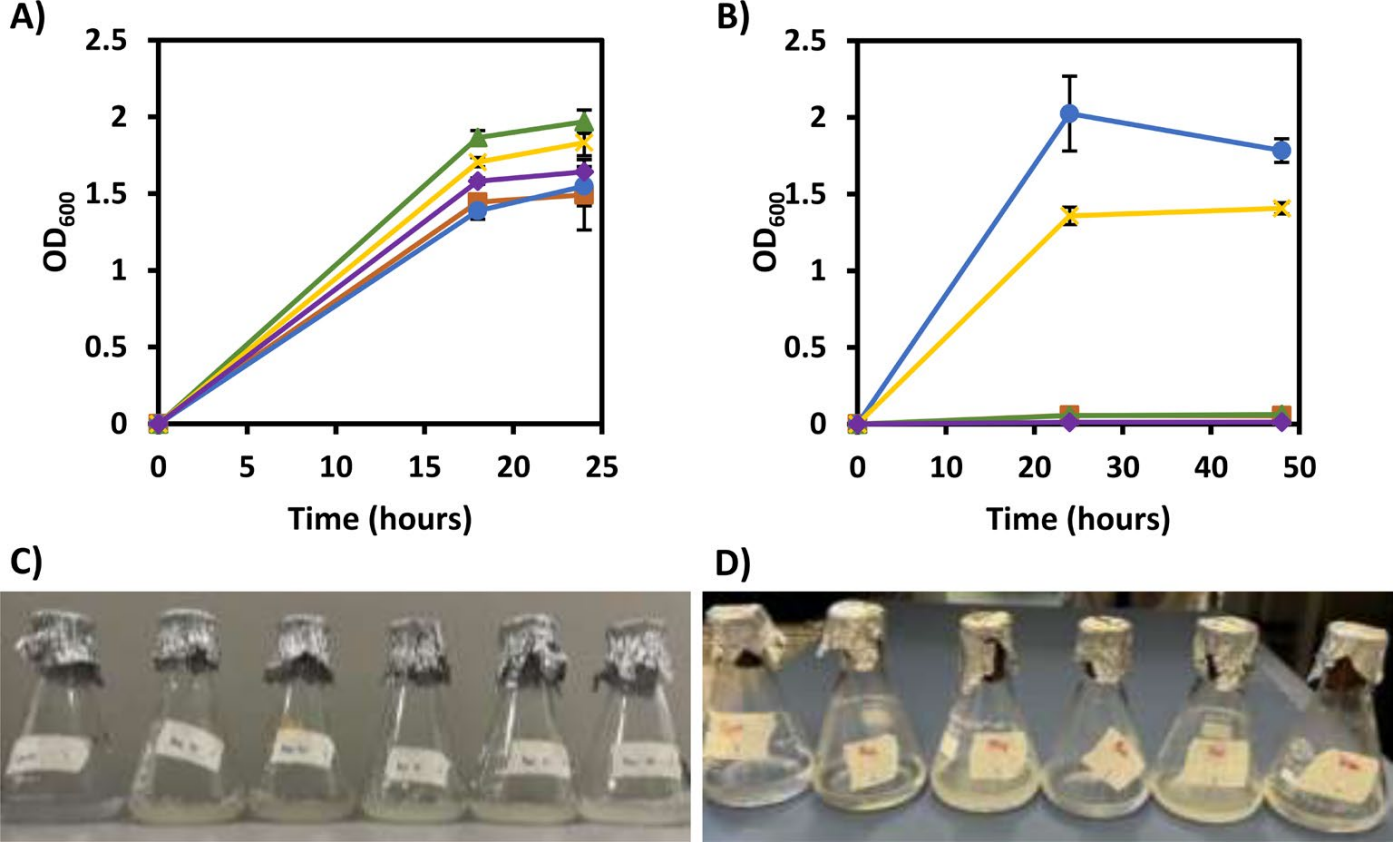
Growth curves for ADP1 (A) WT and (B) M+ in minimal media with acetate (orange), propionate (blue), butyrate (green), valerate (yellow) and hexanoate (purple) over 96 h, grown in biological triplicate. Data points show mean and standard deviation, normalised by subtracting the control with no carbon source. Photographs of 1 flask from each condition for the (C) WT and (D) M+ strain, after 24 and 48 h, respectively.

The ability of ADP1 to grow on VFAs as single carbon sources was tested for C2–C6 straight chain fatty acids with a total carbon concentration of 60 mM. A negative control containing no carbon source was also measured. Figure 2 shows the growth curves for WT ADP1 (Figure 2A,C) and the M+ strain (Figure 2B,D). All VFAs tested were suitable as sole carbon sources for the WT, with maximum growth increasing for longer chain VFAs. In contrast, propionate and valerate were used readily by M+ and in both cases good growth was detected, with maximum OD_600_ of 2.0 ± 0.2 and 1.41 ± 0.04 achieved, respectively. However, cultures on acetate, butyrate and hexanoate showed negligible growth over the period measured.

### 3.2 Propionate and Acetate Tolerances

After being established as a suitable substrate for both strains, propionate tolerance of ADP1 WT and M+ was determined by growth on up to 100 mM propionate (Figure S1). Pre‐culturing on low concentrations of propionate (25 mM) in minimal media was found to improve growth rate and tolerance of both strains, although the effect was much more pronounced for M+. The combined tolerance of both strains to acetate and propionate in a co‐fed culture was also explored, combining acetate concentrations of 25–150 mM with a constant propionate concentration of 25 mM. This time, preculture method made little difference to tolerance, but improved growth rate slightly, indicating acetate poses less of a metabolic challenge to ADP1 than propionate.

### 3.3 Effect of Acetate:Propionate Ratio on Growth and Wax Production

**FIGURE 3:**
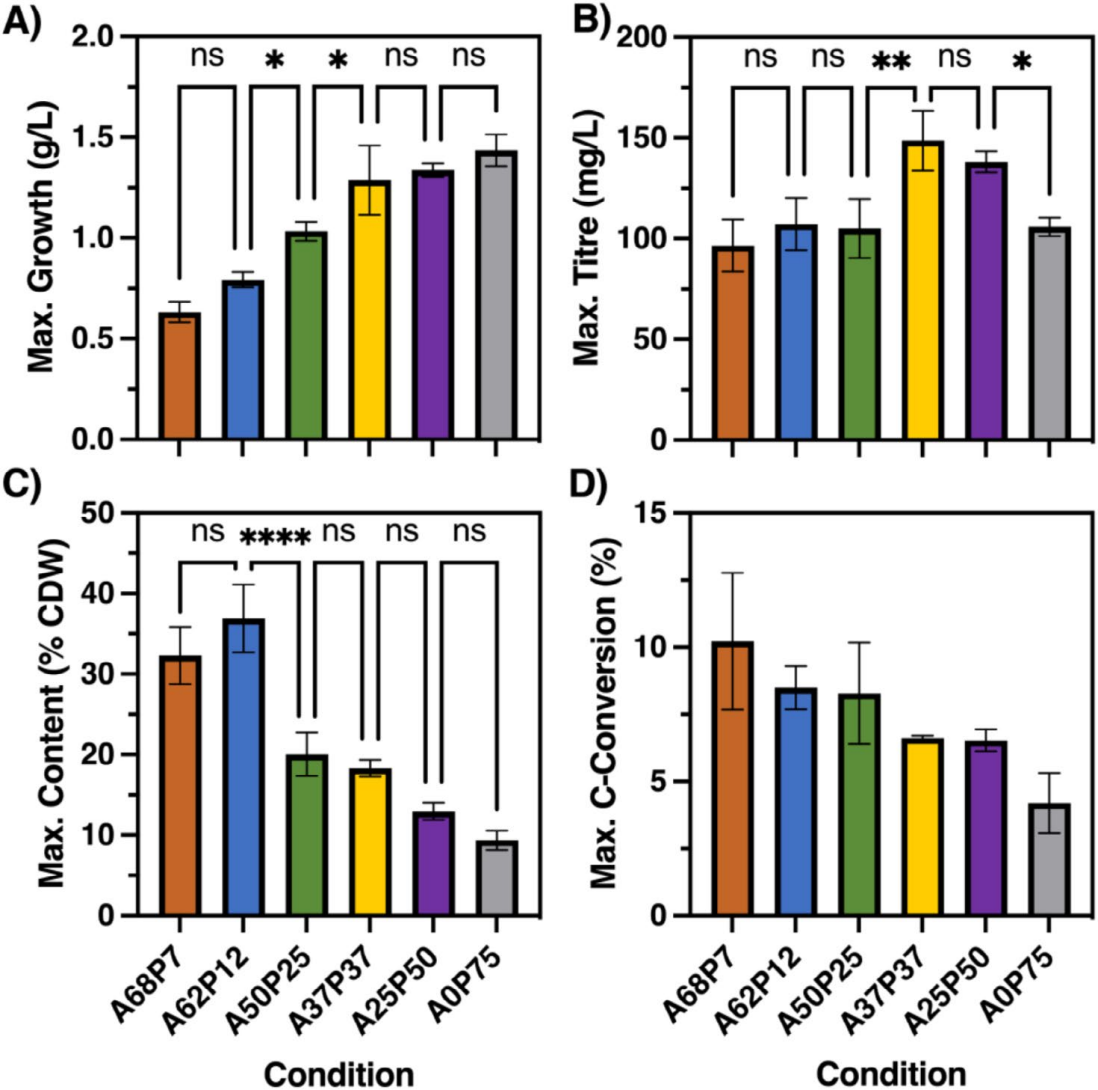
Comparisons of maximum (A) growth, (B) WE titre, (C) WE content per cell and (D) C‐conversion efficiency for ADP1 M+ at different concentrations of acetate (A) and propionate (P), with total acid concentration of 75 mM. Maximums for each metric were taken independently from time course data in Figure S2. Data and error bars represent means and standard deviations, respectively, of three biological replicates. Pairwise significance testing was performed by one‐way ANOVA between sequential conditions, *p* > 0.05 (ns), *p* < 0.05 (*), *p* < 0.01 (**), *p* < 0.0001 (****).

The effect of acetate:propionate ratio on the M+ strain was explored, measuring the effects on both growth and WE production. A total acid concentration of 75 mM was investigated, based on the tolerance tests, with compositions ranging from 68 mM acetate and 7 mM propionate (A68P7) to 75 mM propionate only (A0P75). This resulted in total carbon concentrations from 160 to 225 mM. Cultures were sampled regularly (time course data in Figure S2), and the maximum of each metric for each condition was compared (Figure 3).

As expected, maximum growth increased with propionate proportion (Figure 3A). Maximum titre was comparable at A68P7, A62P12, A50P25 and A0P75, but significantly higher at A37P37 and A25P50 (Figure 3B), with an overall maximum of 150 ± 10 mg/L. Maximum WE content was 37% ± 4% at low propionate proportion (Figure 3C), the highest reported in any bacterial species, although poor growth limited titre. Maximum carbon conversion efficiency also decreased with increasing propionate concentration (Figure 3D), ranging from 10% ± 3% to 4% ± 1%, although pairwise comparisons between adjacent conditions were not significant.

### 3.4 Growth and Wax Production on Acidogenic Fermentate

After confirming acetate–propionate mixtures were suitable for growth and WE production, the suitability of fermentate as a complete growth medium was tested. The AF process utilised *Opuntia ficus indica* (*opuntia*) as a biomass source, a crassulacean acid metabolism (CAM) plant which is adapted for growth in arid conditions due to a specialised photosynthesis pathway that minimises water loss (Snyman 2013). CAM plants are potential bioenergy and bioproduction crops, avoiding competition with agriculture for arable land and growing well in poor soils with erratic rainfall (Yadav et al. 2019), and are a good model for the fermentation of energy crops and agricultural residues. Table 1 shows the concentration of VFAs, nitrogen and phosphate present in the fermentate produced. Fermentate was diluted with deionised water to maintain a constant carbon:nitrogen:phosphate ratio, which is known to impact WE production (Martin et al. 2023).

**TABLE 1:** The composition of the fermentate from *Opuntia ficus indica* used for growth and WE production.

Component	Concentration (g/L)
VFA	20.3 ± 0.1
Acetate	10.2 ± 0.1
Propionate	4.20 ± 0.01
Isobutyrate	0.10 ± 0.02
Butyrate	2.49 ± 0.02
Isovalerate	0.279 ± 0.006
Valerate	1.85 ± 0.01
Hexanoate	1.22 ± 0.04
Total nitrogen	1.15 ± 0.02
Total phosphate	0.191 ± 0.007

**FIGURE 4:**
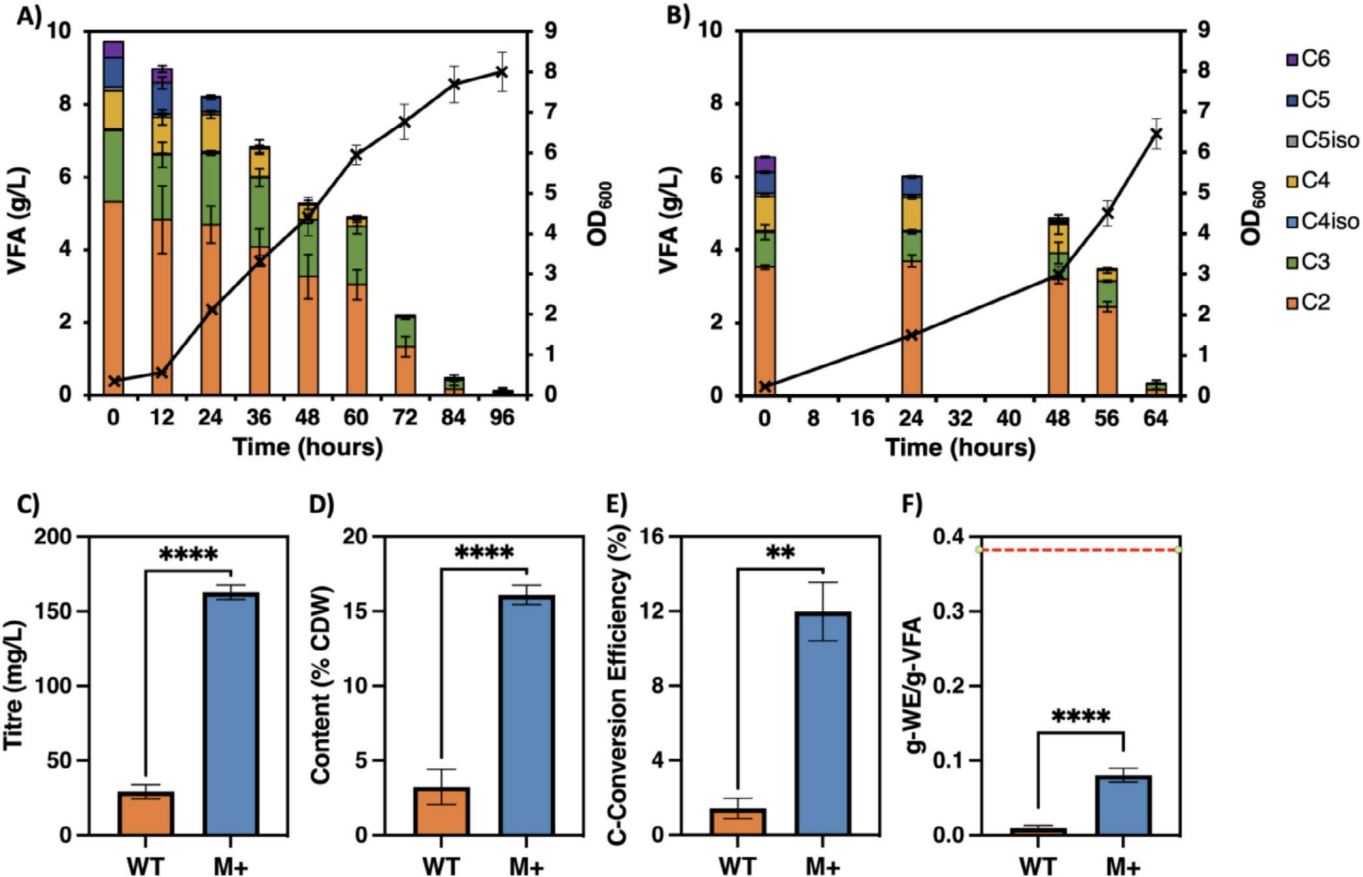
Growth curves and VFA consumption from fermentate for (A) ADP1 WT on 10 g/L total VFA and (B) ADP1 M+ on 6 g/L total VFA. Comparisons of the maximum (C) WE titre, (D) WE content per cell, (E) C‐Conversion efficiency to WE and (F) Mass yield of WE from VFA. The red line indicates the theoretical maximum mass yield from the VFA mixture present in the fermentate. Data and error bars represent the means and standard deviations, respectively, with nine biological replicates for the WT and three biological replicates for M+. Significance of difference between strains analysed by *t*‐test; *p* < 0.01 (**), *p* < 0.0001 (****).

Suitable VFA concentrations were selected for the growth of the M+ and WT strains at 6 g/L VFA and 10 g/L VFA, respectively (see Figure S3). Figure 4A,B show the growth curves and VFA consumption profiles of both strains, measured over 7 days. Growth in both strains was good, with maximum OD_600_ of 8.0 ± 0.4 and 6.5 ± 0.4 for the WT and M+, respectively, and total VFA consumption was observed. WE production was measured at regular intervals, and the maxima achieved for each strain compared (Figure 4C–F). All metrics were significantly higher in M+: titre increased from 29 ± 5 mg/L in the WT to 163 ± 5 mg/L in M+, maximum content from 3% ± 1% to 16.1% ± 0.6%, and C‐Conversion efficiency from 1.4% ± 0.6% to 12% ± 2%. Maximum titre and content per cell were obtained at 36 and 24 h, respectively in the WT bacteria, before decreasing rapidly to near zero. In M+ both were obtained at 48 h.

The total wax yield from VFA consumed (Figure 4F) was 0.009 ± 0.003 g‐WE/g‐VFA in the WT and 0.08 ± 0.01 g‐WE/g‐VFA in M+, calculated from the VFA profiles for each strain at maximum WE titre. The total digestion VFA yield per volatile solids (VS) of 27.7% ± 0.2% for the AF, in agreement with literature values (Tenci et al. 2023), resulted in WE yields from VS of 0.24% ± 0.08% and 2.2% ± 0.3% for WT and M+, respectively.

### 3.5 Increasing Carbon Density Within Volatile Fatty Acid Tolerance Limits

#### 3.5.1 Wax Production Using Longer Chain Volatile Fatty Acids

Growth and production on VFAs are limited by relatively low substrate concentrations used due to the inherent tolerance limits of the bacteria. However, the M+ tolerance limits of acetate and propionate at a range of ratios were similar, evidencing that total acid concentration was a factor. Based on this observation, longer chain fatty acids were investigated as carbon sources, in particular butyrate and valerate, which are also significant components of fermentate.

WE production was explored on combinations of propionate and valerate (growth‐supporting substrates) with acetate and butyrate (production‐supporting substrates) at a range of concentrations and compositions. Two ratios of production‐supporting:growth‐supporting substrates were tested based on the results of the acetate:propionate experiment (Figure 3); 1:1, which gave the highest WE titre, and 5:1, which resulted in the highest WE content per cell. Total acid concentration was also varied to account for the reduced tolerance to butyrate and valerate (Figure S4).

**FIGURE 5:**
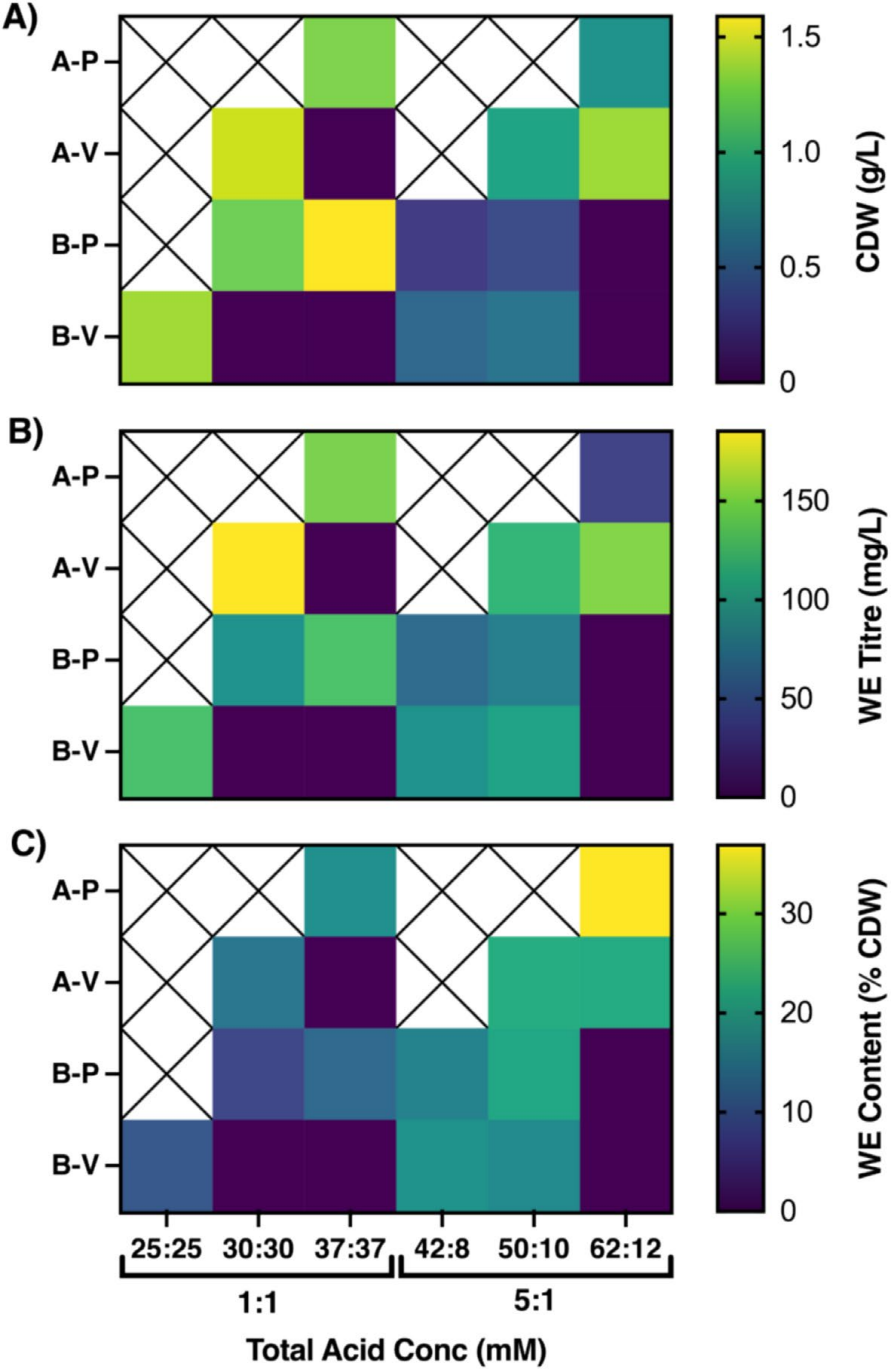
Heat maps of the maximum (A) growth (g/L), (B) titre (mg/L) and (C) content per cell (% CDW) achieved under each condition. Numbers denote concentration of the given acid (25:25 = 25 mM acetate, 25 mM propionate). Crossed out squares indicate conditions not measured due to a low total carbon concentration. Purple squares show samples which did not grow, so all metrics were zero. Data shows mean of three replicates. See Tables S1 and S2 for more details.

With a 1:1 production‐supporting:growth‐supporting substrate ratio, the increased carbon concentration afforded by utilising longer chain VFAs enabled improved growth, up to tolerance limits (Figure 5A and Figure S4). The highest carbon concentration at which growth was achieved was 262.5 mM (see Table S2), with 37.5 mM butyrate and 37.5 mM propionate (B37P37), a 40% increase from the equivalent acetate–propionate experiment. At this concentration, biomass production of 1.6 ± 0.1 g/L was also maximised, an increase of 23% compared to 1.3 ± 0.2 g/L achieved on A37P37. Under 5:1 conditions, growth on 62.5 mM acetate and 12.5 mM valerate (A62V12) was better than A62P12, but growth in butyrate containing cultures was severely limited. Despite B50P10 and B50V10 containing 40% and 54% more carbon than A62P12, growth in both conditions still decreased compared to the acetate–propionate equivalent.

Overall, the trends in WE titre and content were similar to those observed in acetate and propionate cultures (Figure 5B,C). Samples with 1:1 production‐supporting:growth‐supporting substrate ratio grew better than those with a ratio of 5:1, and thus WE titres were higher. Similarly, higher total acid concentrations (up to the tolerance limit) also yielded better growth and higher titres. The A30V30 condition gave the greatest WE titre of 190 ± 10 mg/L, a 27% increase on the maximum achieved in the acetate–propionate experiment. Conversely, WE contents were higher in samples with 5:1 ratio, with a maximum content observed of 23% ± 3%.

#### 3.5.2 Tuning of Fermentate to Replicate Optimal Conditions

Finally, to validate our findings of potential growth and WE titre improvements from longer chain VFAs, AF of dried pig blood was used to generate fermentate with an increased proportion of longer chain VFAs (Tenci et al. 2023). Pig blood has been used previously as a model substrate for high protein biomass (Tenci et al. 2023; Kovács et al. 2013) a common waste stream from animal agriculture which is of interest for anaerobic digestion (Afazeli et al. 2014; Hejnfelt and Angelidaki 2009). A total VFA concentration of 29.8 ± 0.8 g/L was achieved, with absolute and relative composition given in Table 2. C4 and C5 acids, both linear and branched, constituted 50% of the total VFA by mass, a substantial increase from the 23% in the *Opuntia*‐derived fermentate, whereas nitrogen concentration increased 6.4‐fold due to the protein rich feedstock.

**TABLE 2:** The VFA composition of the fermentate obtained by digesting dried pig blood.

Component	Concentration (g/L)	Relative composition (%)
VFA	29.8 ± 0.8	
Acetate	11.1 ± 0.6	37
Propionate	3.5 ± 0.2	12
Isobutyrate	3.4 ± 0.2	11
Butyrate	5.8 ± 0.3	19
Isovalerate	4.8 ± 0.3	16
Valerate	1.1 ± 0.2	3
Hexanoate	0.07 ± 0.03	2
Total organic carbon	17 ± 1	
Total nitrogen	7.4 ± 0.4	

**FIGURE 6:**
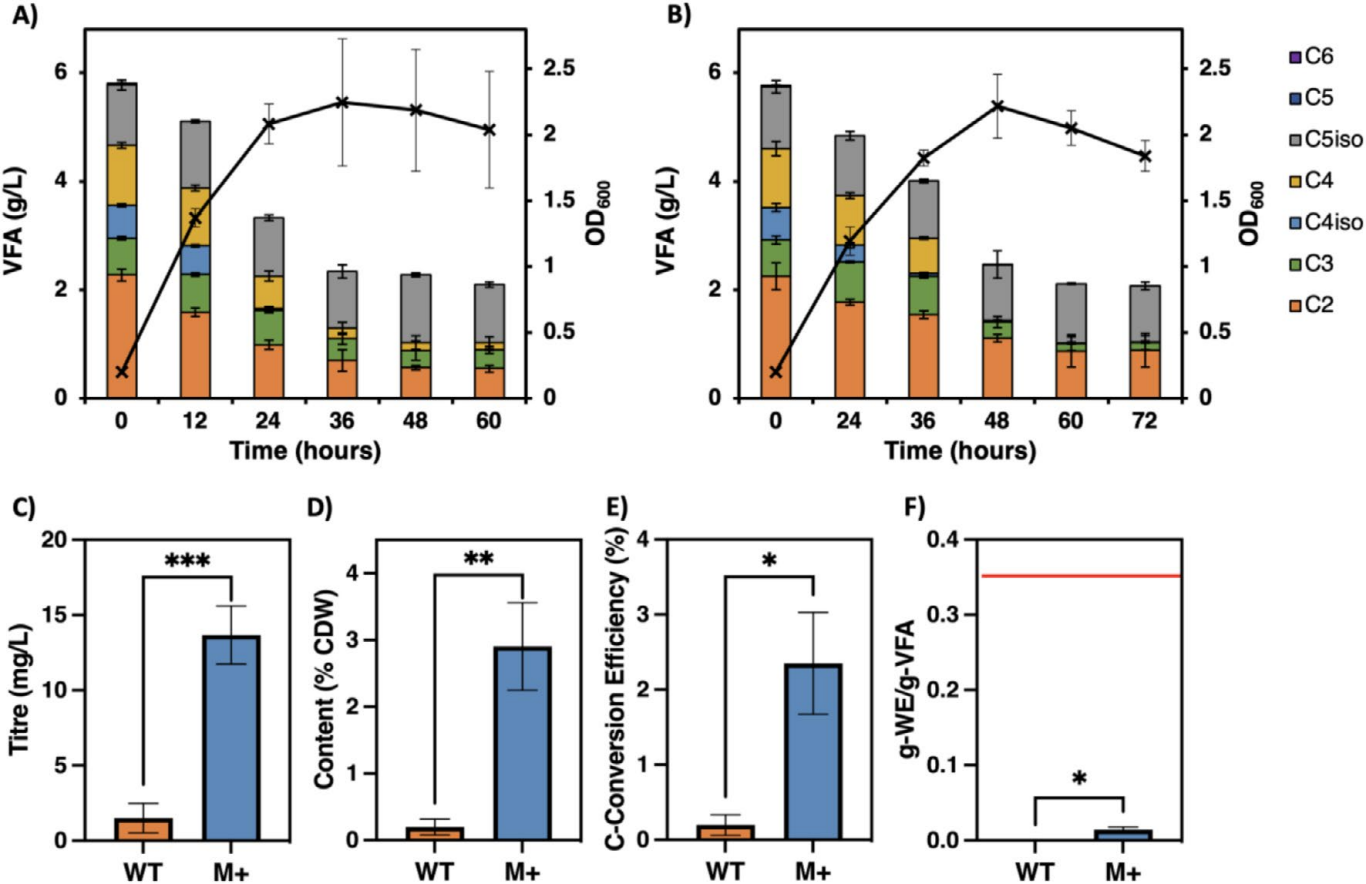
Growth curves and VFA consumption from C4/C5‐rich fermentate for (A) ADP1 WT and (B) ADP1 M+ on 6 g/L total VFA. Comparisons of the maximum (C) WE titre, (D) WE content per cell, (E) C‐Conversion efficiency to WE and (F) Mass yield of WE from VFA. The red line indicates the theoretical maximum mass yield from the VFA mixture present in the C4/C5‐rich fermentate. Data and error bars represent the means and standard deviations, respectively, of three biological replicates. Significance of difference between strains analysed by *t*‐test; *p* < 0.05 (*), *p* < 0.01 (**), *p* < 0.001 (***).

ADP1 WT and M+ were both grown on this C4/C5‐rich fermentate, at a concentration of 6 g/L total VFA. VFA consumption and growth curves are shown in Figure 6A,B for WT and M+, respectively. Both strains grew substantially worse in this media than in the *Opuntia* fermentate, reaching maximum OD_600_ of just 2, and around 2 g/L of VFA remained even after cells entered death phase, including all of the isovalerate, which neither strain was able to utilise. As before, WE production by all metrics was significantly higher in the M+ strain than the WT (Figure 6C–F). Maximum titre, content and C‐conversion efficiency were 14 ± 2 mg/L, 2.9% ± 0.7% CDW and 2.5% in M+, compared to 2 ± 1 mg/L, 0.2% ± 0.1% CDW and 0.2% ± 0.1% in the WT, increases of 9‐, 15‐ and 12‐fold, respectively. WE yield from consumed VFA was also much greater, with 0.014 ± 0.004 in the M+ strain compared to 0.0004 ± 0.0003, a 35‐fold increase. However, absolute values were greatly reduced compared to *Opuntia* fermentate.

## 4 Discussion

### 4.1 Broad Metabolic Findings and Implications

This study has revealed several important genetic and metabolic traits of ADP1. By establishing that the M+ strain can utilise odd‐chain VFAs as sole carbon sources (Figure 2), we confirm that short chain fatty acid metabolism in ADP1 is similar to that of *Escherichia coli*, with propionate metabolised through the methyl‐citrate pathway (Textor et al. 1997). In particular, even‐chain VFAs were broken down into acetyl‐CoA units, whereas odd‐chain VFAs were catabolised into acetyl‐CoA and propionyl‐CoA units (Jaremko and Yu 2011; Rand et al. 2017). This difference in metabolism extended to the requirement for pre‐culturing for metabolic adaption; propionate metabolism is known to be inducible in other species (Brämer et al. 2002), and hence it is unsurprising that ADP1 pre‐adapted to low concentrations of propionate (25 mM) displayed higher tolerance and grew more quickly with shorter lag phase. Interestingly, this benefit was lost with the addition of acetate, behaviour not previously reported or understood. However, this explains the ability of cells to grow in fermentate without a separate pre‐adaptation phase, which would be desirable in large scale processes as it minimises the number of stages, and therefore the number of reactors and the cost, required for cultivation.

### 4.2 Production Without a Production‐Supporting Substrate?

Growth‐supporting:production‐supporting substrate ratio has been previously shown to affect both growth and WE production efficiency of ADP1 (Santala et al. 2021). Here, altering the ratio of propionate:acetate (a simplified version of the available substrates in fermentate) could result in a 4‐fold increase in WE content, a 2.5‐fold increase C‐conversion efficiency and a 50% increase in titre, in agreement with these observations. However, on propionate alone, up to 100 mg/L of WE accumulated even without the addition of the production‐supporting substrate (acetate). In previous studies, ADP1 cultured with gluconate as the growth‐supporting substrate did not accumulate WEs in the absence of acetate despite gluconate‐grown cells generating acetate as a metabolic intermediate, and therefore possessing the inherent capacity for WE synthesis. This suggests WE synthesis under these conditions was not metabolically favoured.

In *E. coli,* propionate has previously been reported to increase production of acetyl‐CoA (Wang et al. 2018) and demonstrated to relieve self‐inhibition of translation of acetyl‐CoA carboxylase (ACC) that catalyses the first step in the fatty acid biosynthesis pathway (Meades et al. 2010). We propose the findings of this work suggest a similar mechanism is present in *A. baylyi* ADP1. Although both propionate and gluconate as growth‐supporting substrates generate acetyl‐CoA, cells grown on propionate may accumulate acetyl‐CoA in a way that gluconate grown cells do not, increasing ACC concentration and hence WE production. This effect would be compounded by flux through the TCA cycle being limited by the glyoxylate shunt knock‐out in the M+ strain and the propionate inhibition of multiple enzymes, another phenomenon observed in *E. coli* (Brock 2010; Stumpf et al. 1980) and which may also apply to ADP1. In addition, metabolism of propionate by the methyl‐citrate pathway increases the concentration of methyl‐citrate propionate grown cells, also shown to activate ACC (Cheema‐Dhadli et al. 1975). These regulatory mechanisms are the likely cause of the observed WE accumulation observed when using propionate as a growth‐supporting substrate, but not with gluconate.

Citric acid cycle equivalents, acetate and citrate, have similarly been shown to downregulate the TCA cycle (Millard et al. 2021) and upregulate ACC (Icard et al. 2021), respectively, in multiple cell types. The greater increase in WE production observed by addition of acetate to cells growing on gluconate compared to propionate is also proposed to be caused by the larger relative change in TCA cycle inhibition and ACC activation.

### 4.3 Potential Benefits of Longer Chain Volatile Fatty Acids

The utilisation of longer chain VFAs (C4–C6) as carbon sources is relatively under‐reported but is of interest for multiple reasons. Fatty acids are catabolised by beta‐oxidation, generating an additional 2 equivalents of NADH per acetyl‐CoA unit cleaved from the backbone, a benefit demonstrated by the initial ADP1 growth test (Figure 2A) where maximum OD was 30% higher on butyrate and valerate than on acetate and propionate, despite equimolar carbon concentrations. Greater reducing power means the maximum theoretical yields of WEs also increase for longer chains: on acetate the maximum is just 0.26 g‐WE/g‐VFA, whereas on butyrate it is 0.42 g‐WE/g‐VFA and on hexanoate it is 0.49 g‐WE/g‐VFA. It was also observed that both the WT and M+ strain preferentially consumed longer chain VFAs over short ones (Figure 4).

Here, a final motivation for utilising longer chain VFAs was to increase total carbon concentration without increasing acid molarity, thus partially circumventing the issue of substrate inhibition. As hoped, increasing chain lengths allowed higher total carbon concentrations without exceeding acid tolerance. However, tolerances were not equal at a molar level because long chains were rapidly metabolised into acetate and propionate units, which were detected in cultures where they were not provided as substrates, causing total acid concentration to temporarily increase. In particular, high concentrations of butyrate reduced growth even when total carbon increased. Replacing acetate with butyrate in VFA mixtures has previously been observed to decrease growth in algal strains (Fei et al. 2015). Similarly, increasing VFA concentration can reduce biomass production, with C4 acids hypothesised to cause acute stress (Moon et al. 2013). In general though, increasing acetyl‐CoA concentration (by directly increasing concentration or by substrate chain elongation) increased biomass production as, despite the glyoxylate shunt knockout, acetate still contributes carbon to the central metabolism (Santala et al. 2021).

### 4.4 Suitability of Engineered Strains for Waste Stream Valorisation

This study is the first to report the production of WEs from a non‐lipid, mixed waste‐stream. Like many organic waste streams (Abbas et al. 2011; Prandi et al. 2019), the abundance of nitrogen in fermentate makes it largely unsuitable for microbial lipid production, which is typically induced by nitrogen‐limitation (Martin et al. 2023; Luo et al. 2020; Wang et al. 2019; Wierzchowska et al. 2021). It is therefore an ideal candidate for growth and bioproduction by metabolically engineered bacteria, in which natural regulatory restrictions can be relaxed. The M+ mutant was specifically chosen for its suitability for wax production from nitrogen‐rich substrates (Luo et al. 2020). However, fermentate does not contain a significant source of glycolytic substrates or amino acids, which were previously required by this strain for growth, and hence its utilisation represents a significant step forward in the biological upgrading of low value waste streams to useful chemicals.

Despite promising results translating growth on propionate and acetate to *Opuntia* fermentate, in the C4/C5 rich fermentate this was not the case: although M+ still significantly outperformed the WT, the absolute values were much lower than in synthetic media. This difference is attributed to the very high ammonium concentration, with a C:N ratio of just 2.7:1, whereas in all the synthetic systems and the *Opuntia* fermentate it was around 10:1. Although the M+ strain was specifically designed to produce higher WE titres and yields in nitrogen‐rich conditions (Luo et al. 2020), it is clear this capability is still limited by natural regulation in ADP1 which prevents WE accumulation at very high nitrogen concentrations.

Two process optimisation approaches exist to increase the C:N ratio of waste streams and overcome this limitation: supply additional carbon or remove the inherent nitrogen. Supplying low concentrations of energy dense substrates like sugars or waste oils offers a combined benefit of increasing the C:N ratio and providing greater reducing and ATP generation potential, which would enhance WE production substantially. Alternatively, decreasing nitrogen concentration is possible using chemical or physical treatments that remove ammonia, the major nitrogen source in fermentate. Gas stripping is widely used in wastewater treatment and reduces the concentration of dissolved ammonia in solution by increasing pH to favour the NH_3_ form and then sparging the solution (Costamagna et al. 2020). In aquaculture and wastewater treatment, the removal of ammonium and other cations is achieved using zeolites (Kumari et al. 2024), porous alumina‐silicate minerals with a very high potential for absorption and exchange of positive ions. Zeolites have recently been employed to increase methane production in anaerobic digestion processes (Wang et al. 2024) by removing ammonium during the fermentation process, but they could also be used as a treatment for acidogenic fermentate pre‐WE production.

Further metabolic engineering and regulatory approaches could also be explored as a potential work‐around for these issues. Nitrogen starvation is linked to the sigma factor sigma‐54 (also referred to as RpoN), which is necessary for transcription of genes involved in nitrogen metabolism and assimilation (Hunt and Magasanik 1985) and was previously found to negatively regulate the glyoxylate shunt in *Pseudomonas aeruginosa* (Hagins et al. 2010), shown here to be critical to WE production. Investigating the role of RpoN in regulating other genes directly or indirectly involved in fatty acid and WE biosynthesis by identifying and locating in the ADP1 genome RpoN consensus sequences, which are known in other bacterial strains (Barrios et al. 1999) could identify other genes of interest for knock‐out or over‐expression mutants.

### 4.5 Towards Theoretical Maximum Yields

The production of highly reduced lipids from highly oxidised VFAs has inherently low yields because cells must generate reducing agents and other necessary co‐factors by the complete oxidation of feedstock to CO_2_, reducing the theoretical maximum efficiency. This is evidenced by the reduction in WE titre demonstrated when using propionate as a growth‐supporting substrate (150 ± 10 mg/L) in comparison to gluconate (340 mg/L; Santala et al. 2021). Although 1 mol of gluconate produces 2 mol of acetyl‐CoA as well as 3 mol of NADH and 1 mol of ATP as cofactors, 2 mol of propionate (which has the equivalent carbon content) also produces 2 mol of acetyl CoA, but only 2 mol of NADH. When yield is considered in terms of mass, this effect is compounded by the mass of oxygen present in VFAs that is lost in their conversion to WEs.

An estimate of the theoretical maximum yield in fermentate was calculated from the VFA consumption profile of the M+ strain at maximum WE production. This maximum of 0.37 ± 0.02 g‐WE/g‐VFA (Figure 4F) accounted for the necessary carbon, reducing equivalents (NADPH) and ATP for WE production only, and did not consider other cellular functions such as biomass production, which would further reduce this value. The yield achieved in M+ of 0.08 ± 0.01 g‐WE/g‐VFA was therefore at least 22% of the theoretical maximum yield, highlighting the potential of utilising engineered bacteria for bioproduction to efficiently valorise complex, mixed waste streams.

### 4.6 Practical Challenges and Implications of Using Real Fermentate

One of the major benefits of whole cell lipid biosynthesis compared to chemical or biocatalytic approaches is the adaptability of the bacterial growth to variable substrate input. However, as evidenced in Figure 5, the growth and WE production potential are significantly affected by VFA composition. This presents a significant challenge for scale‐up, since AF feedstocks are seasonally and geographically variable. Furthermore, given the current state of the literature in the AF field, knowledge of how VFA generation efficiency and composition varies between substrates and conditions is limited due to inconsistent reporting of units, titres and yields.

Mitigating the potential negative impacts of this will only be possible by improving the data availability. Studies should focus on varying the parameters of the AF fermentation to achieve the broadest possible range of VFA compositions. The possible concentration range for each VFA could then be treated as an independent variable within a design of experiments study to investigate how they interact. Machine learning models may also aid in extracting optimal condition combinations from such datasets (Helleckes et al. 2023). Once target conditions are identified, a second round of AF parameter exploration should be undertaken, this time with a goal of producing and maintaining the optimal VFA mixture across a range of feedstocks by controlling process parameters (pH, temperature, retention time, organic loading, and inoculum source).

Another issue facing the scale‐up of AF for bioproduction is process intensity. In many applications, such as wastewater and sewage treatment, the concentrations of AF substrates, and hence of the generated VFAs, are low, typically a few grams per litre (Amulya et al. 2016). Large culture volumes and energy‐intensive dewatering steps make the capital and operational expenditure for such processes at industrial scale prohibitively expensive. Utilising concentrated waste streams, such as fermentate from digestions with high substrate loading as investigated here, which are suitable for fed‐batch and continuous culture processes, allows greater biomass accumulation and hence higher WE titres. Similarly, doping fermentate with more reduced, higher energy waste streams such as waste cooking oils or crude glycerol also facilitates denser bacterial growth and more concentrated product streams, which are cheaper and more efficient to handle, extract and purify.

## 5 Conclusions

The results obtained here represent a significant step towards the utilisation of VFAs, in particular VFA‐rich waste streams, as sole carbon and energy sources for WE production. By utilising an engineered strain of ADP1, WE titres of up to 190 mg/L were achieved on synthetic VFAs and up to 160 mg/L on fermentate, with a 5.6‐fold increase over the WT. The highest ever WE content per cell in ADP1 of 37% CDW was also recorded.

However, the challenges of translating a process from synthetic media conditions to a complex, mixed substrate were also highlighted; in particular, the difficulty of obtaining a waste stream with an optimal, controllable or even predictable composition. Mapping the global VFA landscape with respect to AF process conditions and feedstock composition will be necessary to achieve reliable bioproduction processes from this abundant, renewable carbon source. Advances in product intensification by increasing substrate energy density are also undoubtedly essential in improving the process scalability and economic viability of industrial bioproduction of microbial lipids.

## Author Contributions

**Ian P. Thompson:** funding acquisition, project administration, resources, supervision, writing – review and editing. **Wei E. Huang:** supervision. **Laura K. Martin:** investigation, methodology, formal analysis, writing – original draft, writing – review and editing, validation, visualization.

## Funding

This study was supported by the Engineering and Physical Sciences Research Council (EP/T517811/1 and D4T00370‐DF04.01).

## Conflicts of Interest

The authors declare no conflicts of interest.

## Supporting information


**Figure S1:** Growth curves and tolerance tests for ADP1 WT (A–D) and M+ (E–H) grown on propionate and acetate in minimal media.
**Figure S2:** Comparisons of the (A) growth, (B) WE titre, (C) WE content per cell and (D) C‐conversion efficiency for ADP1 M+ mutant over time at different concentrations of acetate (A) and propionate (P), with total acid concentration of 75 mM in all conditions.
**Figure S3:** Growth curves for *Acinetobacter baylyi* ADP1 M+ on VFA rich digestate at a range of dilutions.
**Figure S4:** Growth curves obtained in 96 well plates for ADP1 M+ grown in minimal media on varying concentrations and combinations of acetate, propionate, butyrate and valerate.
**Table S1:** Summary table of the different combinations and concentrations of acetate (A), propionate (P), butyrate (B) and valerate (V) tested for WE production and the total carbon concentration under each condition.
**Table S2:** Maximum growth (g/L of cell dry weight) of ADP1 M+ on different concentrations and combinations of acetate, propionate, butyrate and valerate.

## Data Availability

The data that support the findings of this study are available from the corresponding author upon reasonable request.
