# Supplementary material for: Mixed Waste Streams for Bioproduction: Exploring Bacterial Wax Ester Production in Nitrogen‐Rich Acidogenic Fermentate
Source: Microb Biotechnol. 2026 Feb 16;19(2):e70314. doi: 10.1111/1751-7915.70314 (PMC12908686; doi:10.1111/1751-7915.70314)
Supplement: Supplementary file 1 — Figure S1: Growth curves and tolerance tests for ADP1 WT (A–D) and M+ (E–H) grown on propionate and acetate in minimal media. Figure S2: Comparisons of the (A) growth, (B) WE titre, (C) WE content per cell and (D) C‐conversion efficiency for ADP1 M+ mutant over time at different concentrations of acetate (A) and propionate (P), with total acid concentration of 75 mM in all conditions. Figure S3: Growth curves for Acinetobacter baylyi ADP1 M+ on VFA rich digestate at a range of dilutions. Figure S4: Growth curves obtained in 96 well plates for ADP1 M+ grown in minimal media on varying concentrations and combinations of acetate, propionate, butyrate and valerate. Table S1: Summary table of the different combinations and concentrations of acetate (A), propionate (P), butyrate (B) and valerate (V) tested for WE production and the total carbon concentration under each condition. Table S2: Maximum growth (g/L of cell dry weight) of ADP1 M+ on different concentrations and combinations of acetate, propionate, butyrate and valerate. [file MBT2-19-e70314-s001.zip › mbt270314-sup-0001-FigureS1-S4-TableS1-S2@Mixed waste streams for bioproduction SI Corrections.docx]

## Supplementary information


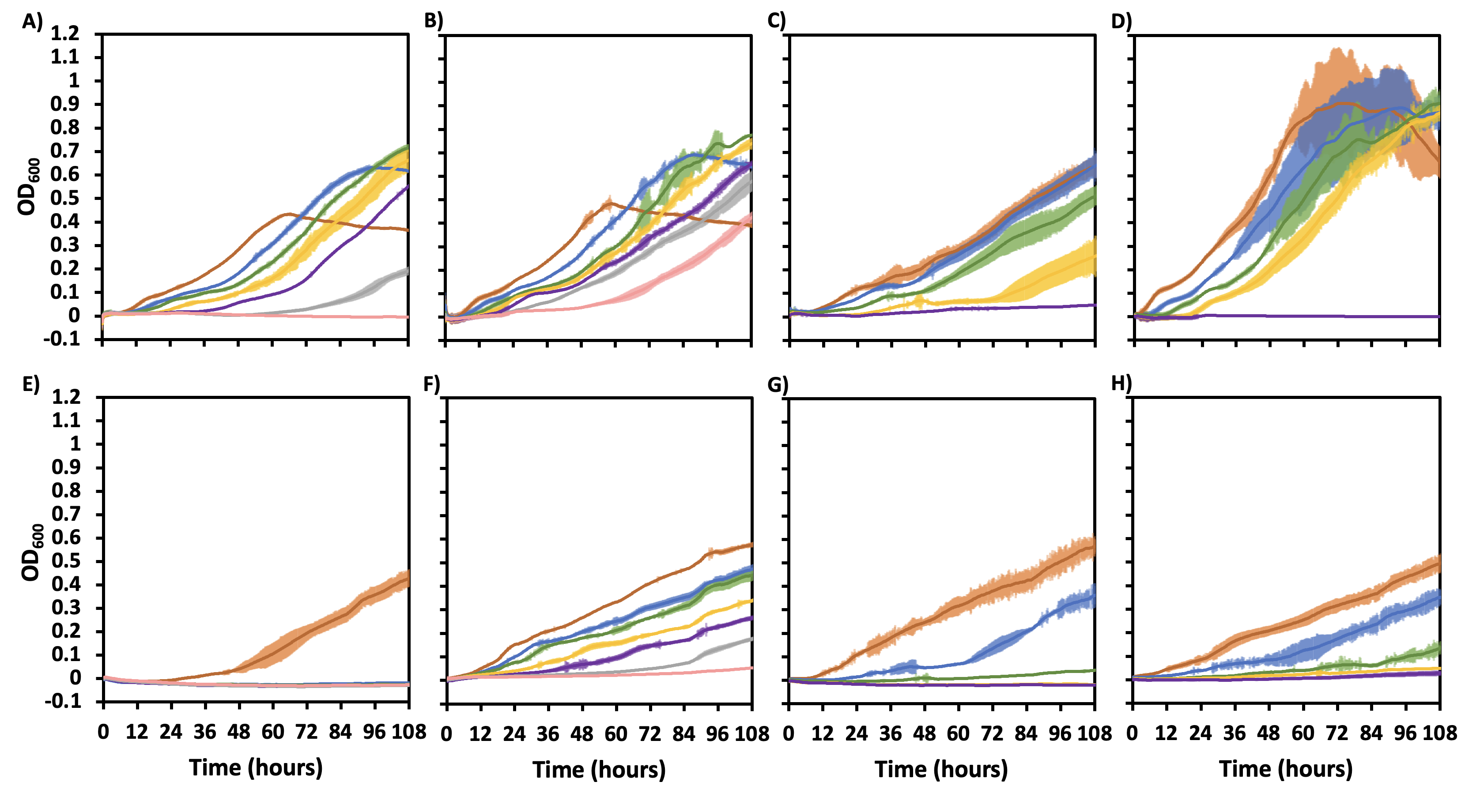


**Figure S1.** Growth curves and tolerance tests for ADP1 WT (A-D) and M+ (E-H) grown on proprionate and acetate in minimal media. A), B), E) and F) cells cultured on propionate at 25 mM (orange), 37.5 mM (blue), 50 mM (green)l 62.5 mM (yellow), 75 mM (purple), 87.5 mM (grey) and 100 mM (pink). C), D), G) and H) cells cultured on 25 mM propionate with acetate at 25 mM (orange), 50 mM (blue), 75 mM (green), 100 mM (yellow) and 150 mM (purple). Cultures in A), C), E) and G) were precultured on LB, while those in B), D), F) and H) were precultured on 25 mM propionate in minimal media. Data and error bars represent the means and standard deviations, respectively, of three biological replicates. Means were smoothed by adjacent averaging over 20 points. Negative controls of precultured cells with no carbon source were used as a blank.


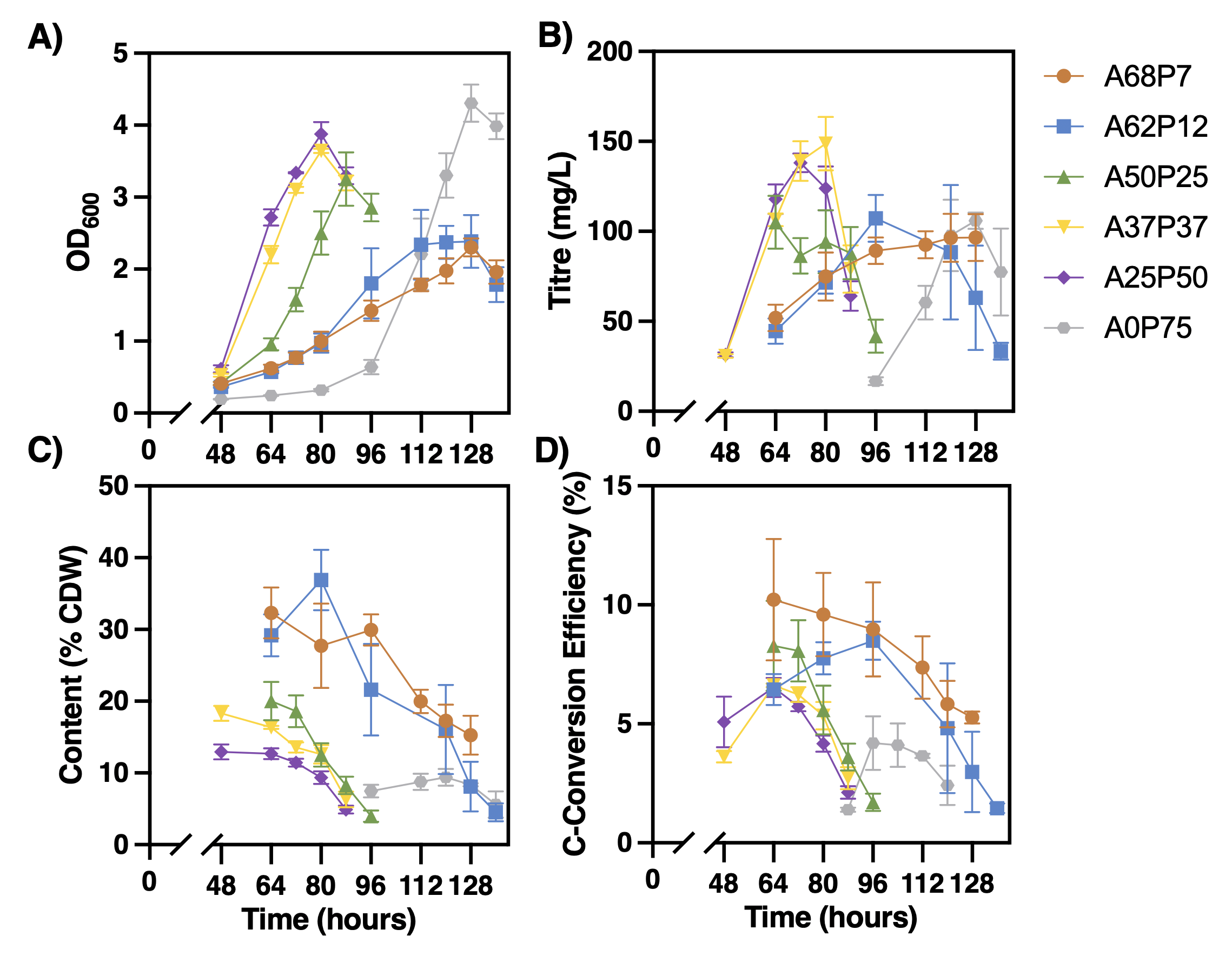


**Figure S2.** Comparisons of the A) growth, B) WE titre, C) WE content per cell and D) C-conversion efficiency for ADP1 M+ mutant over time at different concentrations of acetate (A) and propionate (P), with total acid concentration of 75 mM in all conditions. Numbers denote concentration of the given acid (A68P7 = 68 mM acetate, 7 mM propionate). Data and error bars represent the means and standard deviations, respectively, of three biological replicates. Conditions were A68P7 (orange circles), A62P12 (blue squares), A50P25 (green triangles), A37P37 (yellow inverse triangles), A25P50 (purple diamonds), A0P75 (grey circles).

**Figure S3.** Growth curves for *A. baylyi* ADP1 M+ on VFA rich digestate at a range of dilutions. VFA concentrations of 4 (orange), 6 (blue), 8 (green), 10 (yellow) and 12 g/L (purple) were tested.


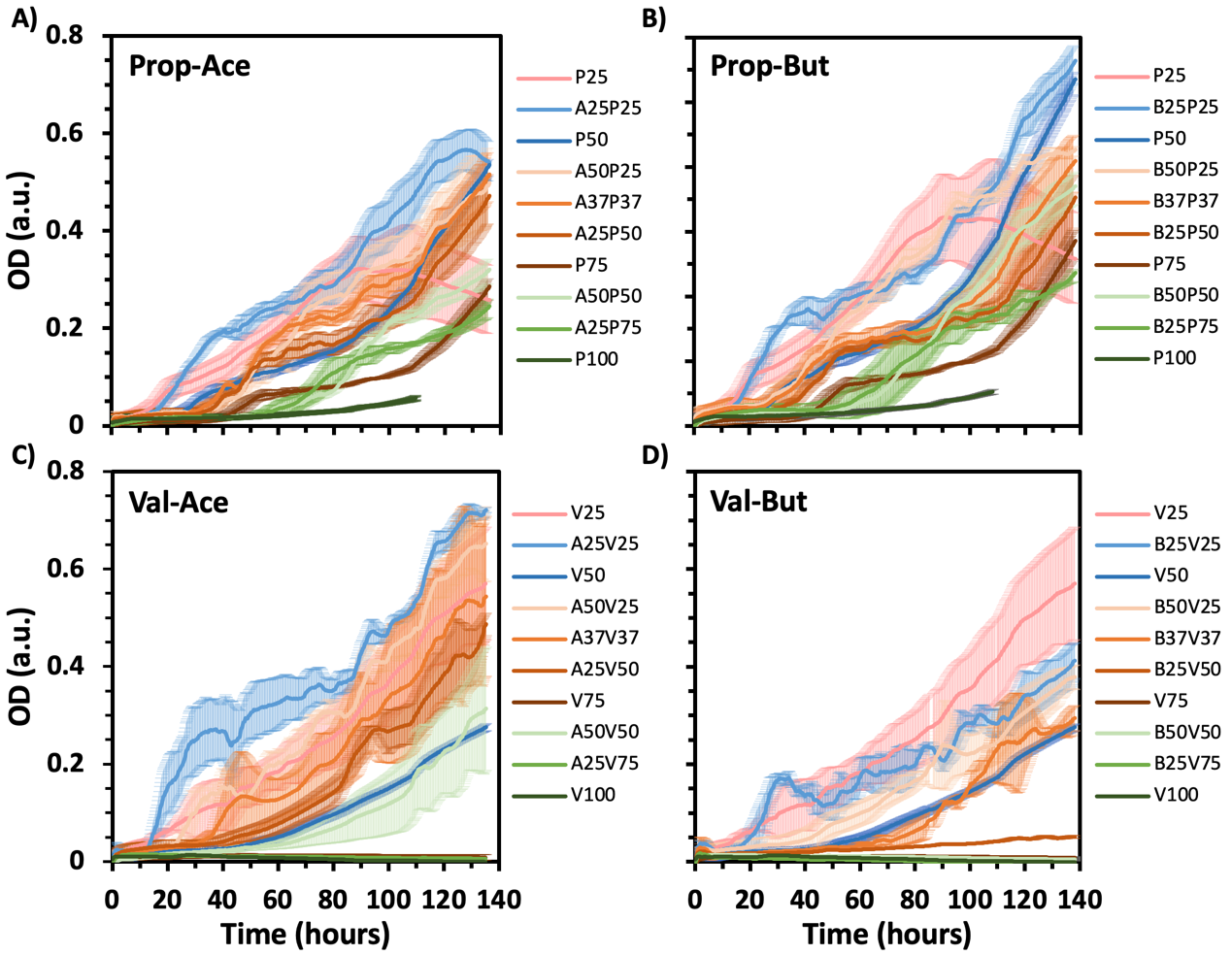


**OD_600_**

**OD_600_**

**Figure S4.** Growth curves obtained in 96 well plates for ADP1 M+ grown in minimal media on varying concentrations and combinations of acetate, propionate, butyrate and valerate. A) Propionate-Acetate, B) Propionate-Butyrate, C) Valerate-Acetate and D) Valerate-Butyrate. Data and error bars represent the means and standard deviations, respectively, of three biological replicates.

**Table S1.** Summary table of the different combinations and concentrations of acetate (A), propionate (P), butyrate (B) and valerate (V) tested for WE production, and the total carbon concentration under each condition. Samples containing high concentrations of valerate (37.5 mM) or butyrate (above 62.5 mM), shown in bold, and were unable to grow (also see Figure 6A). In conditions underlined one of the three replicates failed to grow and WE production data was calculated from the two grown replicates.

| **VFA Ratio** | **1:1** | | | **5:1** | | |
| --- | --- | --- | --- | --- | --- | --- |
| **Total VFA (mM)** | **50** | **60** | **75** | **50** | **60** | **75** |
| **VFAs** | **Total Carbon Concentration (mM)** | | | | | |
| A-P | - | **-** | 187.5 | - | **-** | 162.5 |
| A-V | - | 210 | **262.5** | - | 150 | 187.5 |
| B-P | - | 210 | 262.5 | 191.7 | 230 | **287.5** |
| B-V | 225 | **270** | **337.5** | 208.3 | 250 | **312.5** |

**Table S2.** Maximum growth (g/L of cell dry weight) of ADP1 M+ on different concentrations and combinations of acetate, propionate, butyrate and valerate.

| **VFAs** | **Total Carbon Concentration (mM)** | | | | | |
| --- | --- | --- | --- | --- | --- | --- |
|  | **1:1** | | | **5:1** | | |
|  | **50** | **60** | **75** | **50** | **60** | **75** |
| A-P | - | - | 1.3 ± 0.2 | - | - | 0.8 ± 0.2 |
| A-V | - | 1.47 ± 0.05 | 0 | - | 0.9 ± 0.1 | 1.4 ± 0.3 |
| B-P | - | 1.24 ± 0.07 | 1.6 ± 0.1 | 0.28 ± 0.04 | 0.36 ± 0.01 | 0 |
| B-V | 1.4 ± 0.1 | 0 | 0 | 0.52 ± 0.04 | 0.61 ± 0.04 | 0 |
